# Investigating microbiota and biochemical changes in vaginal fluid toward point-of-care microbial monitoring using surface-enhanced Raman spectroscopy

**DOI:** 10.1117/1.BIOS.2.4.042102

**Published:** 2025-07-25

**Authors:** Anna S. Rourke-Funderburg, Viannely A. Francisco, Dalton J. Nelson, Kate L. Goncalves, Frederick R. Haselton, Emad Elsamadicy, Andrea K. Locke

**Affiliations:** a Vanderbilt University, Department of Biomedical Engineering, Nashville, Tennessee, United States; b Vanderbilt University, Vanderbilt Biophotonics Center, Nashville, Tennessee, United States; c Vanderbilt University Medical Center, Division of Maternal Fetal Medicine, Department of Obstetrics & Gynecology, Nashville, Tennessee, United States; d Vanderbilt University, Department of Chemistry, Nashville, Tennessee, United States

**Keywords:** surface-enhanced Raman spectroscopy, vaginal health, microbiome, vaginal fluid, bacteria

## Abstract

**Significance:**

Vaginal health is maintained by the vaginal microbiome, and dysbiosis of this community can have lifelong negative consequences for women. Current clinical techniques for detecting bacteria in the vagina rely on subjective visual and microscopic analysis or untimely microbial culturing. Surface-enhanced Raman spectroscopy (SERS), a biochemical fingerprinting technique, shows potential for filling this gap as it can identify bacterial species and strains.

**Aim:**

In this study, SERS was used to investigate biochemical changes in vaginal fluid when common vaginal bacteria were present and absent. Subsequently, the performance of a portable Raman spectrometer to detect these biochemical changes was evaluated.

**Approach:**

Vaginal fluid was collected from participants attending routine gynecology exams, and SERS spectra were collected using a Raman microscope and a portable spectrometer. Partial least squares, peak intensity, and peak ratio analysis were used to investigate biochemical differences. Quantitative polymerase chain reaction was performed for characterization of *Lactobacillus iners*, *Lactobacillus crispatus*, *Gardnerella vaginalis*, and *Streptococcus agalactiae* content.

**Results:**

*Gardnerella vaginalis* presence was characterized by a significant increase in protein and lipid-related features and a decrease in organic acid peaks. The presence of *Lactobacillus iners* was represented by increased organic acid peaks and a reduction of protein, amino acid, and polysaccharide-related features. Similar trends with little loss of significance were observed when comparing the performance of a Raman microscope and a portable spectrometer.

**Conclusion:**

We highlight the feasibility of SERS for detecting differences in bacterial species presence in vaginal fluid and showcase the potential for clinical translation.

Statement of Discovery This work uses surface-enhanced Raman spectroscopy to characterize biochemical features related to the presence and absence of key vaginal microbes in vaginal fluid.

## 1 Introduction

The native bacteria that inhabit the vagina play an important role in the defense of the reproductive system from pathogens and make up part of the vaginal microbiome.^1^^–^^3^ In most women, this bacterial community is predominantly composed of *Lactobacillus* species with low microbial diversity. These *Lactobacillus* species protect the vagina from pathogenic growth by producing antimicrobial compounds, including lactic acid, hydrogen peroxide, and bacteriocins.^4^^–^^7^ The four common vaginal *Lactobacillus* species include *Lactobacillus iners* (*L. iners*), *Lactobacillus crispatus* (*L. crispatus*), *Lactobacillus gasseri* (*L. gasseri*), and *Lactobacillus jensenii* (*L. jensenii*). However, *L. iners* has been reported to be the most prevalent species in the vaginal microbiome.^8^^–^^11^ When there is a loss of dominance of vaginal *Lactobacillus*, this is known as vaginal dysbiosis and can have many negative effects for women throughout their lifetime. During pregnancy, vaginal dysbiosis can increase the risk of preterm birth, premature rupture of membranes, and neonatal sepsis.^4^^,^^12^ Outside of pregnancy, vaginal dysbiosis can increase the risk of acquiring sexually transmitted infections (STIs) and urinary tract infections (UTIs), lead to the development of yeast infections, reduce the conception rate for women undergoing *in vitro* fertilization, and may play a role in the development of cervical cancer.^4^^,^^13^^,^^14^ Bacterial vaginosis (BV) is the most common form of vaginal dysbiosis and is characterized by a replacement of *Lactobacillus* species by anaerobic bacteria. BV is a polymicrobial infection, but *Gardnerella vaginalis* (*G. vaginalis*) is the most commonly identified pathogen detected during BV, being detected in up to 95% of cases.^6^^,^^15^ Another prevalent vaginal pathogen, *Streptococcus agalactiae* (*S. agalactiae*), also known as Group B *Streptococcus*, is associated with various dysbiosis conditions and can cause significant complications when found in high concentration during pregnancy and labor and in nonpregnant populations with co-morbidities, such as diabetes and cirrhosis.^16^^–^^19^

Although *L. iners* is reportedly the most prevalent *Lactobacillus* species in the vaginal microbiome, current clinical techniques used for detecting vaginal bacteria are not optimal for detecting this organism. The Nugent method, currently considered the gold standard, relies on Gram staining followed by visual analysis for bacterial morphotypes.^20^ *L. iners* was originally reported as Gram-positive, yet numerous studies have reported this species staining Gram variable or Gram-negative.^8^^,^^10^^,^^21^ A study by Yoshimura et al.^22^ even showed *L. iners* switching its Gram status at various points during culturing. In addition, *L. iners* has been observed with varying cellular morphology. Although *L. iners* was first reported to have bacillary (rod-shaped) morphology, in accordance with the *Lactobacillus* genus, recent studies have shown coccobacillary (short rod or oval) morphology.^8^^,^^21^^,^^22^ As the Nugent criteria relies on Gram staining and cellular morphology to determine if vaginal dysbiosis is present, the variable characteristics of *L. iners* can result in the misidentification of this species. This could lead to inaccuracies in the calculation of the Nugent score and may contribute to the modest sensitivity (∼60%) and specificity (∼80%) of this technique.^23^ Outside of the issues posed by the unique characteristics of *L. iners*, the Nugent method is highly subjective, leading to variability when using this method.^24^ Culturing of vaginal fluid is also used to determine the presence and identity of vaginal bacteria.^25^ *L. iners* lacks the capability to grow on de Man–Rogosa–Sharpe (MRS) agar, which is used for culturing *Lactobacillus* species.^8^^,^^21^^,^^26^ Furthermore, it has been reported that positive *L. iners* cultures can be confused with positive *G. vaginalis* cultures.^11^ These unique characteristics of *L. iners* reduce the likelihood of accurate identification. This may lead to inaccurate diagnoses of vaginal dysbiosis, highlighting the need for more robust techniques to monitor the health of the vaginal microbiome. Molecular-based methods are gaining popularity and show good sensitivity and specificity but are still limited by the need for trained technicians, the probability of contamination, and their complexity.^24^

Vibrational spectroscopies, namely Raman spectroscopy and surface-enhanced Raman spectroscopy (SERS), show promise in providing an accurate technique for detecting differences in vaginal microbiome composition associated with dysbiosis.^27^ Raman spectroscopy and SERS are non-invasive, non-destructive, and can provide results in real time, making them promising methods for rapid, culture-free detection of bacteria in biofluids.^28^ Raman spectroscopy, an inelastic scattering technique, provides information on the biochemical composition of a sample.^29^ Although Raman spectroscopy has been applied to biomedical applications due to the high specificity of its measurement, its sensitivity is limited by the inherent weakness of the Raman scattering phenomenon. SERS can increase the signal strength of conventional Raman spectroscopy while retaining biochemical specificity. SERS can enhance Raman scattering up to 10 orders of magnitude by placing analytes in close proximity (2–10 nm) to colloidal metallic nanoparticles or roughened metallic surfaces.^30^ By leveraging the biochemical specificity of Raman spectroscopy and enhancing its sensitivity, SERS can discriminate microbes at the species and strain levels based solely on their vibrational spectra.^31^^,^^32^ Therefore, SERS is a promising alternative for the detection of bacteria in biological fluids.

Previous research utilizing SERS to study vaginal fluid is limited (i.e., <10 research articles). The first published study of SERS of vaginal fluid investigated changes to the biochemical composition of vaginal fluid throughout the menstrual cycle; this study also presented preliminary analysis from six participants with active vaginal infections and showed that distinct spectral variation was found when a vaginal infection was present.^27^ Following this work, two other studies investigated the SERS spectra of vaginal fluid samples with differing levels of vaginal fluid purity/cleanliness and vulvovaginal candidiasis infection.^33^^,^^34^ Finally, a study by Wen et al.^35^ used SERS coupled with machine learning to determine if BV was present. Although these studies demonstrate the applicability of SERS to measure biochemical signals in vaginal fluid related to vaginal health status, they are limited in the amount of clinical information provided. Previous studies utilized Gram staining to determine the microbes present and grouped all *Lactobacillus* species together at the genus level. Literature has shown that the protective capabilities of the four main *Lactobacillus* species can vary, so knowing the specific species present could facilitate better clinical care.^11^ Furthermore, these studies detect dysbiosis-related bacterial species following clinical diagnosis, but early detection of these microbes would be beneficial as many dysbiosis conditions present with similar symptoms or are asymptomatic.^36^^,^^37^ Therefore, an investigation into the use of SERS to detect specific *Lactobacillus* species, such as *L. iners*, and to detect dysbiosis-associated bacteria prior to clinical diagnosis is warranted.

Herein, the goals of this pilot study were twofold. The first goal was to investigate spectral differences in relation to the presence and absence of common vaginal bacteria in vaginal fluid from healthy participants. The second goal was to examine the use of a portable Raman spectrometer to assess spectral changes in vaginal fluid to highlight the point-of-care applicability of this technique. We utilized quantitative polymerase chain reaction (qPCR) to determine and quantify the presence of *L. iners*, *L. crispatus, G. vaginalis*, and *S. agalactiae.* The qPCR results showed that *L. iners* and *G. vaginalis* were the most prevalent species in our samples, and the SERS spectra were grouped based on qPCR results. Spectral trends within each group were analyzed to determine differences related to the presence and absence of *L. iners* and *G. vaginalis*. To the best of our knowledge, this work demonstrates the first use of SERS to investigate the influence of the presence and absence of *L. iners* and *G. vaginalis* on biochemical changes in vaginal fluid.

## 2 Methods

### 2.1 Materials

Proteose peptone, (4-(2-hydroxyethyl)-1-piperazineethanesulfonic acid) (HEPES), heat-inactivated horse serum, yeast extract, dextrose, lysozyme, brain heart infusion (BHI) powder, MRS media, MRS agar, and BD BBL CultureSwab EZ swabs were purchased from Fisher Scientific (Hampton, New Hampshire, United States). Agar powder, TRIS hydrochloride, Triton X-100, and ethylenediaminetetraacetic acid (EDTA) were purchased from Sigma-Aldrich (St. Louis, Missouri, United States). Luna Universal qPCR Master Mix (catalog # M3003L) was purchased from New England Biolabs (Ipswich, Massachusetts, United States). *L. iners* (strain AB107, ATCC #55195), *L. crispatus* (strain: VPI 7635, ATCC #33197), *G. vaginalis* (strain 594 [NCTC 10287], ATCC #14018), and *S. agalactiae* (strain: NCTC 8181 [G19], ATCC #13813) were purchased from the American Type Culture Collection (ATCC) (Manassas, Virginia, United States). Primers for qPCR were synthesized by Integrated DNA Technologies (Coralville, Iowa, United States). Standard aluminum foil (Reynolds Wrap; Reynolds, Lake Forest, Illinois, United States) was purchased from a local supermarket. All chemicals were of analytical grade and used with no further purification.

### 2.2 Participant Recruitment and Sample Collection

This study was approved by the Vanderbilt University Medical Center Institutional Review Board (IRB #222199). Overall, 19 participants (Table S1 in the Supplementary Material) who attended annual gynecology appointments were recruited for this study using written informed consent. A total of 38 vaginal swabs were collected and analyzed. Vaginal fluid samples were collected from each participant following the placement of a sterile speculum. Two sterile swabs (BD BBL™ CultureSwab EZ) were held in the posterior fornix of the vagina for 10 s sequentially. Swabs were stored on ice until they were transported to the laboratory for processing within <6  h. The end of each swab was cut and placed into 500  μL of sterile deionized water and centrifuged at 3000 g for 5 min to elute the vaginal fluid from the swab. After elution, the swab was discarded, and the solution was aliquoted and stored at −80°C until analysis.

### 2.3 SERS of Vaginal Fluid

Citrate-capped gold nanoparticles (AuNPs) with a diameter of 36 nm (surface charge = −48 mV) were synthesized for this study following previously published methods.^38^ Vaginal fluid aliquots were allowed to thaw fully, and a 2μL droplet of vaginal fluid was applied to an aluminum foil–covered microscope slide and allowed to dry. Then, a 2  μL droplet of AuNPs (1.4 nM) was added on top of the dried vaginal fluid droplet and allowed to dry. Spectra were collected from each sample on two Raman systems. First, SERS spectra were collected using a benchtop Renishaw inVia Qontor Raman microscope (Renishaw, Gloucestershire, United Kingdom) with an excitation wavelength of 785 nm, a 1200  l/mm grating, and a spectral resolution of $1\;{\mathrm{cm}}^{-1}$. Point spectra were collected under 20× magnification (NA = 0.4) with ∼7 to 8 mW laser power measured at the sample and a total integration time of 30 s. Under 20× magnification, the laser spot is rectangular with approximate dimensions of 56×9  μm. Spectra from the same dried sample on the aluminum foil–covered microscope slide were then collected using a Wasatch WP-785X-F18-R-ILC portable Raman spectrometer (Wasatch Photonics, Morrisville, North Carolina, United States) equipped with a fiber optic probe (NA = 0.22, 105  μm fiber core diameter) and a 785 nm excitation laser. Point spectra were collected with 180 mW power at the sample and a total integration time of 10 s. The portable spectrometer has a circular beam profile with a diameter of 168  μm and $7\;{\mathrm{cm}}^{-1}$ spectral resolution. Additional specifications for each system are detailed in Table S2 in the Supplementary Material. For both systems, five spectra were collected from each droplet, and three droplets were measured for each vaginal fluid sample.

### 2.4 DNA Extraction of Bacteria and Vaginal Fluid

For DNA extraction from the four bacteria (*L. iners*, *L. crispatus*, *G. vaginalis*, and *S. agalactiae*), each was cultured according to procedures included in the Supplementary Material. Standard curves for the quantification of each bacterium in the vaginal fluid samples were generated using the cultured ATCC bacteria. DNA was extracted from cultured bacteria and vaginal fluid samples using the QIAamp DNA Mini Kit (Qiagen, Hilden, Germany) according to the manufacturer’s recommendations. Extracted DNA was eluted in Buffer AE (supplied in the QIAamp DNA Mini Kit) and stored at −20°C.

### 2.5 Polymerase Chain Reaction

Following DNA extraction, qPCR was used to determine and quantify the presence of *L. iners*, *L. crispatus*, *G. vaginalis*, and *S. agalactiae* in vaginal fluid samples using previously published parameters.^21^^,^^39^ Real-time fluorescence was monitored using Luna Universal qPCR Master Mix. For each assay, the forward and reverse primer sequences are listed in Table S3 in the Supplementary Material. For all assays, each 20  μL PCR contained 1× Luna Universal qPCR Master Mix, the forward and reverse primers at working concentration (Table S3 in the Supplementary Material), and 2  μL of the extracted DNA. Three-step PCR was performed for the *L. iners, G. vaginalis*, and *S. agalactiae* assays following the cycling conditions listed in Table S3 in the Supplementary Material. Two-step PCR was performed for the *L. crispatus* assay following the cycling conditions listed in Table S3 in the Supplementary Material. The PCRs were performed with a Rotor-Gene Q (Qiagen, Hilden, Germany), and samples that produced amplification within <45 cycles were considered test positive. Each reaction was performed in triplicate and included a standard curve of bacterial DNA from cultures ranging from $1\times 10^{3}$ to $1\times 10^{9}\;\mathrm{CFU}/\mathrm{mL}$ and a no-target control containing molecular grade distilled water. Automated quantification cycle (Cq) determination and amplification analysis were performed with the Rotor-Gene Q Series software version 2.0.3. The standard curve for each bacterium was utilized to determine the concentration of each organism in each vaginal fluid sample by fitting a linear trend line to the standard curve and calculating the unknown bacterial concentration using the Cq and this curve.

### 2.6 Spectral Processing, Analysis, and Statistical Analysis

All spectra were smoothed using a second-order Savitsky–Golay filter (window size = 7).^40^ The background fluorescence was subtracted from each spectrum using asymmetric least squares regression (Renishaw: λ=15000, p=0.001; Wasatch: λ=20000, p=0.001).^41^ Finally, spectra were normalized using standard normal variate normalization.^42^ For analysis, the five spectra collected from each droplet were averaged together (Fig. S1 in the Supplementary Material). The average spectra were grouped into three groups based on the bacterial presence as determined by qPCR (Table S4 in the Supplementary Material). Dimensionality reduction was performed using partial least squares (PLS) on the average spectra from each droplet.^43^ All spectra were truncated to 600 to $1675\;{\mathrm{cm}}^{-1}$ prior to dimensionality reduction. The model was trained using leave-one-participant-out cross validation, which leaves all the spectra from a certain participant out while training the model and then applies the model to the left-out spectra. The variable important in projection (VIP) was calculated using the PLS weights and amount of variance explained by each predictor to determine spectral features that contributed significantly to the model.^43^^,^^44^ Peaks highlighted as important in the VIP plot were further analyzed for significant differences among the groups using peak intensity and peak ratio analysis. Kruskal–Wallis tests and *post hoc* Dunn’s multiple comparison tests were performed to compare the significance of the peak intensities and ratios among the groups. An alpha value of 0.05 was used for all statistical analyses. All spectral processing, PLS, and peak ratio calculations were performed using MATLAB (MathWorks, Natick, Massachusetts, United States). Statistical analyses were performed using MATLAB and GraphPad Prism 10 (GraphPad Software, San Diego, California, United States). Figure generation and spectral plotting were performed in Origin (OriginLab, Northampton, Massachusetts, United States). All data are presented as mean ± standard deviation, except where otherwise indicated.

## 3 Results

The SERS spectra of vaginal fluid collected from participants seeking routine gynecological care were characterized and analyzed in three groups based on bacterial presence identified via qPCR. Participant demographics can be found in Table S1 in the Supplementary Material. Kruskal–Wallis tests were used to determine if participant demographics varied significantly among the three groups. We found that age, race, sexual intercourse within two weeks, and contraceptive use did not significantly differ among the three groups (p=0.46, 0.26, 0.27, and 0.12, respectively).

**Fig. 1:**
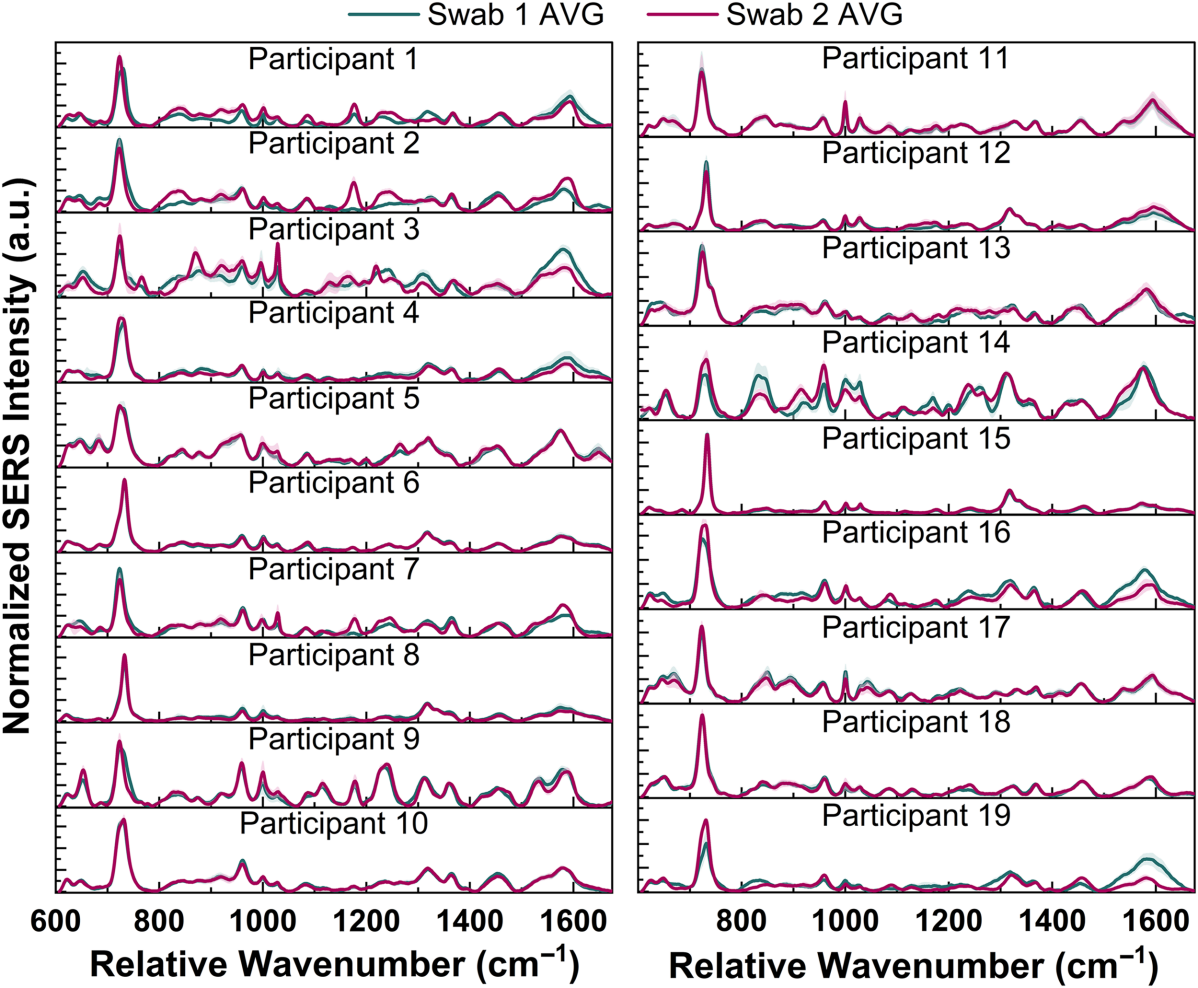
SERS spectra of vaginal fluid eluted from swabs collected from 19 participants. Each spectrum is the mean and standard deviation (represented as shaded error bars) for the three droplets measured for each swab (n=15 spectra/swab, 2 swabs/participant).

As measured by qPCR, 32 of the 38 swabs collected in this study were found to contain *Lactobacillus* at concentrations consistent with the reported bacterial concentration of the vaginal microbiome ($10^{7}$ to $10^{9}\;\mathrm{CFU}/\mathrm{mL}$).^45^^,^^46^ Twelve swabs did not contain any quantifiable amount of *L. iners* or *G. vaginalis* (Table S4 in the Supplementary Material). Figure 1 shows the average SERS spectra collected from each vaginal fluid sample in this study. Variations in the spectral profile are seen among the different participants. All spectra collected are dominated by a peak between 723 and $734\;{\mathrm{cm}}^{-1}$, which is commonly reported to be from the bacterial cell wall, stemming from adenine-containing compounds (NAD, FAD, DNA, etc.) or peptidoglycan in the cell wall.^31^^,^^47^^–^^49^ Other peaks noted in the SERS spectra arise from well-known vaginal fluid components, including organic acids [lactic, acetic, citric] (850, 880–890, 920, 1085, and 1595–$1640\;{\mathrm{cm}}^{-1}$), proteins and amino acids (622, 650, 885, 850, 880–890, 920, 935, 960, 1000, 1028, 1175, 1240, 1290, 1305–1315, 1360, 1440–1470, and 1575–$1640\;{\mathrm{cm}}^{-1}$), lipids (960, 1028, 1290, 1305–1315, and 1440–$1470\;{\mathrm{cm}}^{-1}$), carbohydrates [monosaccharides and polysaccharides] (880–890, 935, 960, and $1028\;{\mathrm{cm}}^{-1}$), and urea ($1000\;{\mathrm{cm}}^{-1}$) (Table S5 in the Supplementary Material).

We found low spectral variability between the two swabs collected per participant for most participants (Fig. 1). However, variation is seen in spectral profile in a few regions for certain participants, such as the 1500 to $1625\;{\mathrm{cm}}^{-1}$ region for participant #3 and the 800 to $1100\;{\mathrm{cm}}^{-1}$ region for participant #14. To account for this variability, we treated each swab as an individual sample rather than averaging the spectra collected from each participant.

**Fig. 2:**
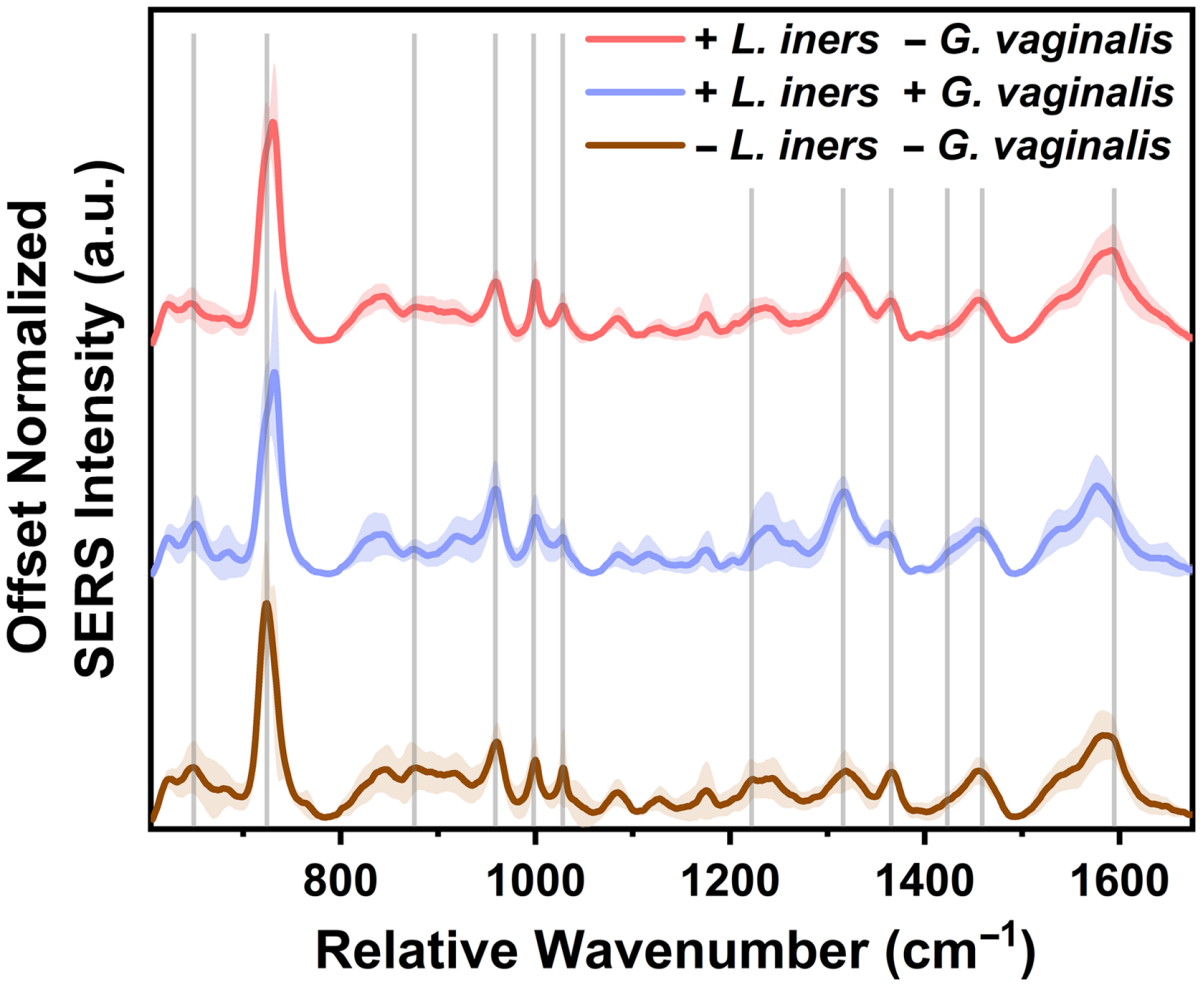
SERS spectra of vaginal fluid grouped by bacteria detected in each swab. The average and standard deviation (represented as shaded error bars) of all spectra per group are presented. Gray lines represent features that varied among the three groups. +LI/−GV = 14 swabs (n=41 spectra), +LI/+GV = 12 swabs (n=36 spectra), and −LI/−GV = 12 swabs (n=36 spectra). LI = *L. iners*. GV = *G. vaginalis*.

The SERS spectra collected from each swab were grouped based on bacterial presence as determined by qPCR. *S. agalactiae* was not detected in any of the samples collected. *L. iners* and *G. vaginalis* were the most prevalent species detected from the samples collected, so the groupings were designed based on the presence and absence of these microbes. In 14 swabs, corresponding to seven participants, *L. iners* was detected, but no *G. vaginalis* was detected [+*L. iners*, −*G. vaginalis*]. *L. iners* and *G. vaginalis* [+*L. iners*, +*G. vaginalis*] were detected in 12 swabs, representing six participants. *L. iners* and *G. vaginalis* [–*L. iners*, −*G. vaginalis*] were not detected in 12 swabs (six participants) (Table S4 in the Supplementary Material). The average SERS spectra for each group are plotted in Fig. 2. To investigate the variability within groups, the coefficient of variation (standard deviation/mean) was calculated using the peak intensities at $727\;{\mathrm{cm}}^{-1}$. The coefficient of variation is lowest for the group where only *L. iners* was detected at 12.82%. The other two groups have higher coefficients of variation, ranging from 19% to 23%. Despite this in-group variability, spectral variation is also seen among the three groups characterized by varying peak intensities (Fig. 2). Variations can be seen in spectral features reported to arise from protein and amino acids (650, 960, 1000, 1028, 1240, 1315, 1360, 1440–1450, and 1595–$1640\;{\mathrm{cm}}^{-1}$). Features relating to lipid content (960, 1028, 1315, and 1440–$1450\;{\mathrm{cm}}^{-1}$) also differ among the three groups. Finally, peaks relating to organic acids and carbohydrate content (890, 1028, and 1595–$1640\;{\mathrm{cm}}^{-1}$) also vary among the groups.

**Fig. 3:**
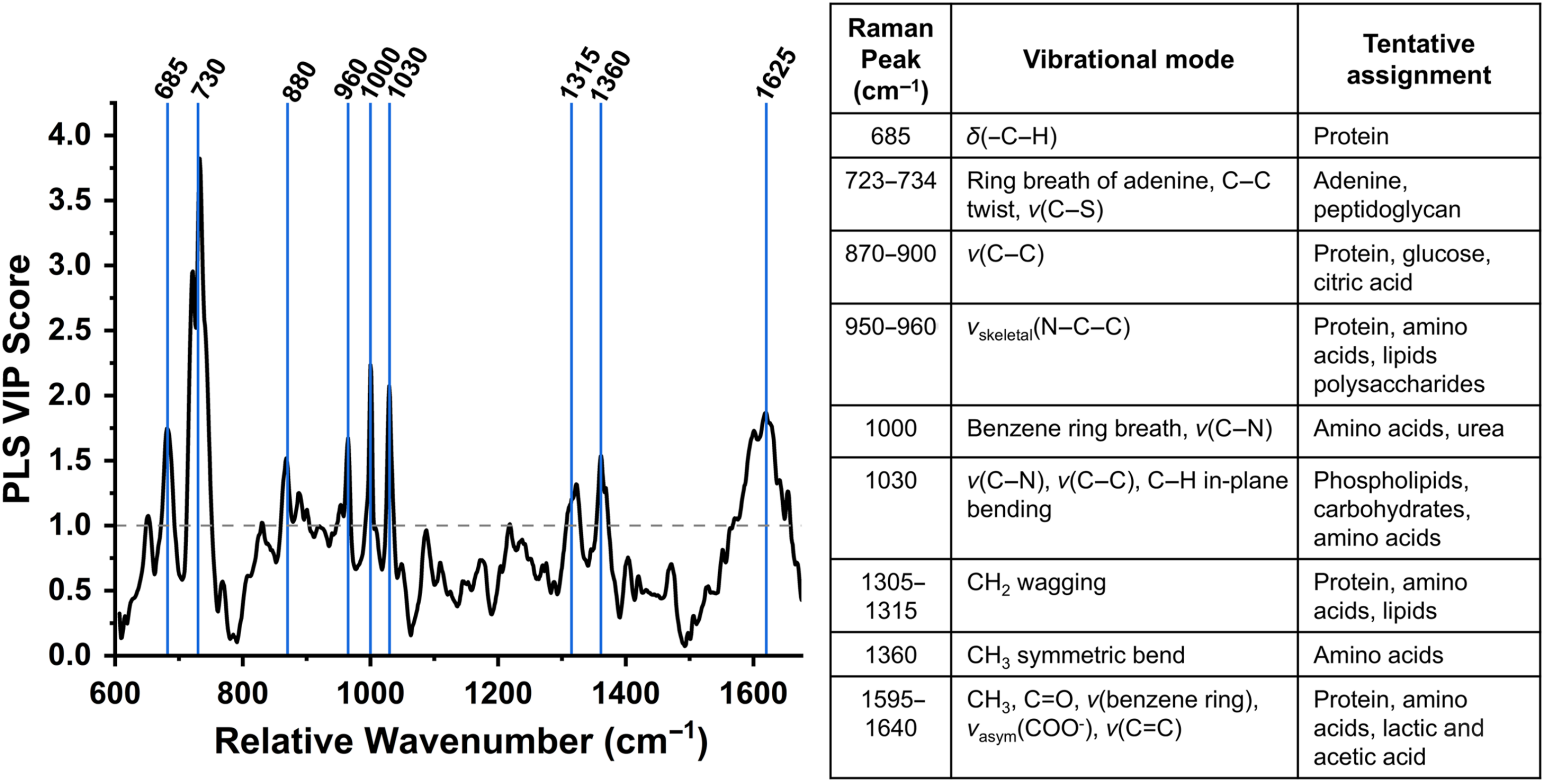
Variable importance in projection (VIP) plot highlighting features with a score greater than 1.0, indicating importance, and table of vibrational mode and tentative biochemical assignments for important features. (ν = stretching, δ = deformation, twist = twisting, sciss = scissoring, sym = symmetric, asym = asymmetric, breath = breathing).

Dimensionality reduction was performed via PLS. The resultant VIP plot was utilized to determine spectral features that significantly contribute to the model performance (Fig. 3). Nine regions of interest were identified as having a VIP score>1.0. These features are representative of biochemical molecules, including proteins and amino acids, organic acids (such as lactic, acetic, and citric acid), polysaccharides, peptidoglycan, and lipids. Further statistical analysis was performed to identify significant trends in these features using peak ratio and peak intensity analysis.

**Fig. 4:**
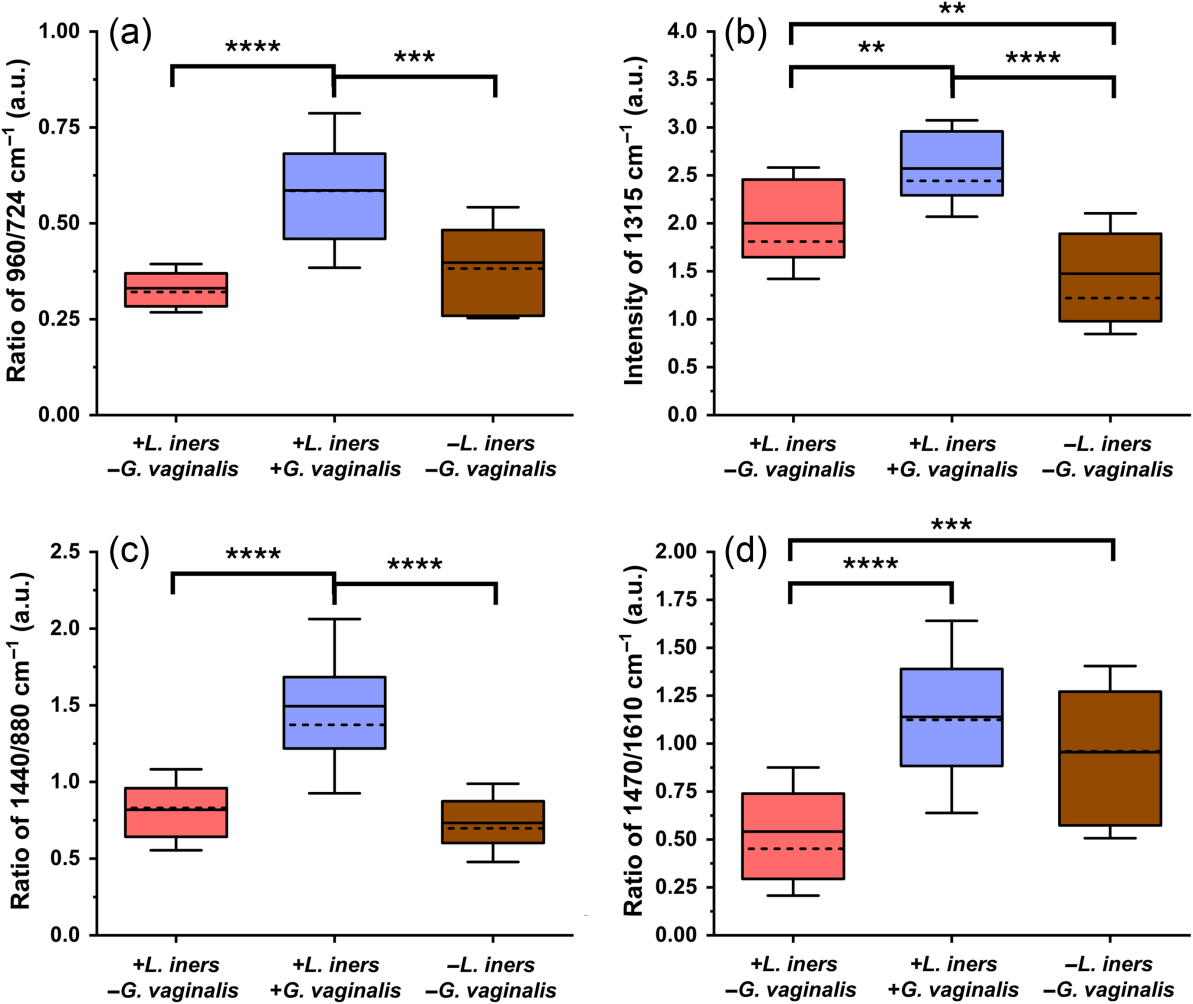
Box plots showing peak intensity and peak ratio analysis: (a) ratio of $960/724\;{\mathrm{cm}}^{-1}$ (protein and lipids/adenine and peptidoglycan); (b) intensity of $1315\;{\mathrm{cm}}^{-1}$ peak (proteins, amino acids, and lipids); (c) ratio of 1440/880 cm^−1^ (lipids); (d) ratio of $1470/1610\;{\mathrm{cm}}^{-1}$ (lipids); box = 25–75 percentile, whiskers = standard deviation, solid line = mean, dashed line = median. +LI/−GV = 14 swabs (n=41 spectra), +LI/+GV = 12 swabs (n=36 spectra), and −LI/−GV = 12 swabs (n=36 spectra). **p<0.01, ***p<0.001, ****p<0.0001. LI = *L. iners*. GV = *G. vaginalis*.

The peak intensity and peak ratio analyses show that the presence of *L. iners* and *G. vaginalis* results in significant biochemical changes in the SERS spectra. First, we examined the ratio of protein and lipid content in relation to cell wall features, as represented by the ratio of $960/724\;{\mathrm{cm}}^{-1}$ (Table S5 in the Supplementary Material). When *G. vaginalis* is present in the vaginal fluid, this peak ratio is significantly higher than when it is not present in the fluid [Fig. 4(a)]. In addition, when *G. vaginalis* is present, there is a significant increase in the peak intensity at $1315\;{\mathrm{cm}}^{-1}$ [Fig. 4(b)], which is reported to be associated with relative protein, amino acids, and lipid content. To further investigate the lipid content, we utilized the ratio of $1440/880\;{\mathrm{cm}}^{-1}$. This ratio shows a significant increase when *G. vaginalis* is present [Fig. 4(c)]. We also found that when *L. iners* is present without *G. vaginalis* [+*L. iners*, −*G. vaginalis*], there is a significant decrease in the relative lipid content, as shown using the ratio of $1470/1610\;{\mathrm{cm}}^{-1}$, as compared with when *L. iners* is present with *G. vaginalis* [+*L. iners, +G. vaginalis*] and not present [−*L. iners*, −*G. vaginalis*] [Fig. 4(d)].

**Fig. 5:**
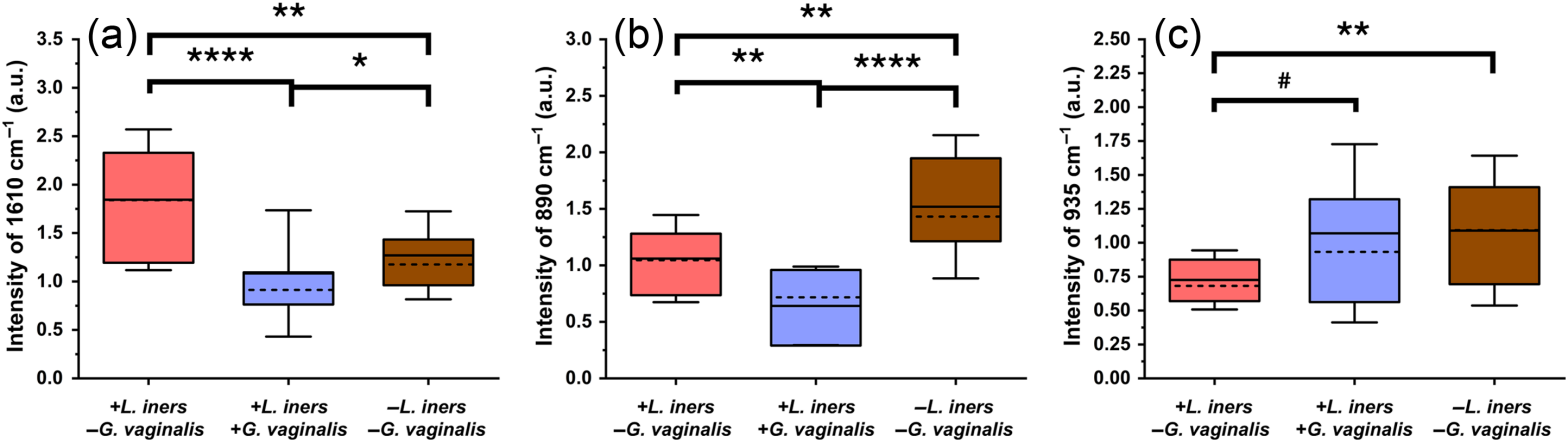
Box plots showing peak intensity analysis: (a) intensity of $1610\;{\mathrm{cm}}^{-1}$ peak (organic acids); (b) intensity of $890\;{\mathrm{cm}}^{-1}$ peak (organic acids); (c) intensity of $935\;{\mathrm{cm}}^{-1}$ peak (protein, amino acids, and polysaccharides); box = 25–75 percentile, whiskers = standard deviation, solid line = mean, dashed line = median. +LI/−GV = 14 swabs (n=41 spectra), +LI/+GV = 12 swabs (n=36 spectra), and −LI/−GV = 12 swabs (n=36 spectra). *p<0.05, **p<0.01, ***p<0.001, ****p<0.0001, #p=0.06. LI = *L. iners*. GV = *G. vaginalis*.

**Fig. 6:**
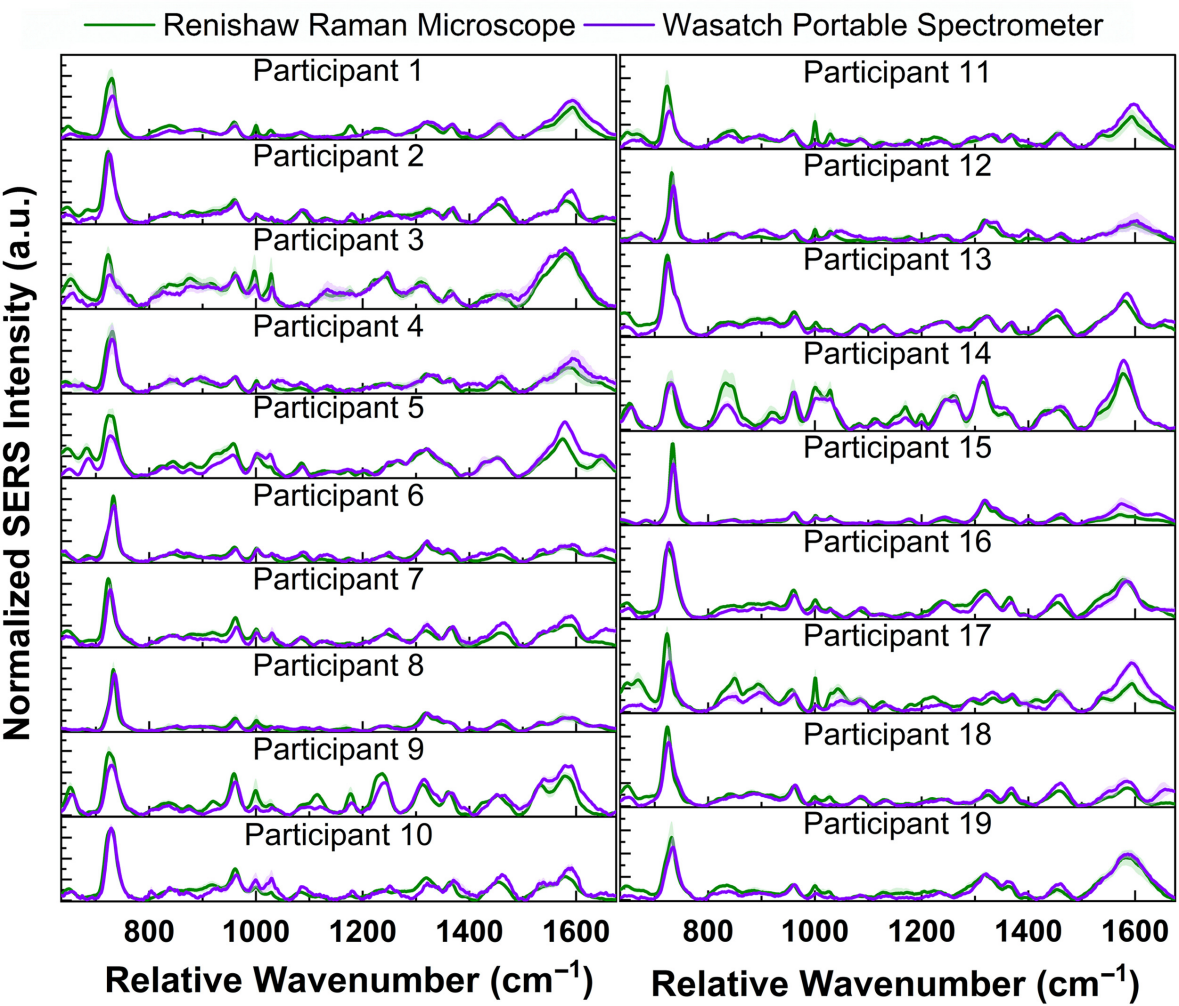
Comparison of vaginal fluid spectra collected from each participant using the Renishaw Raman microscope (green line) and Wasatch portable spectrometer (purple line). Each spectrum is the mean and standard deviation (represented as shaded error bars) for the three droplets measured from swab 1 from each participant (n=15 spectra/swab).

Next, organic acid content was evaluated when *G. vaginalis* was present and compared with when *L. iners* was and was not detected, by examining the intensities at $890\;{\mathrm{cm}}^{-1}$ and $1610\;{\mathrm{cm}}^{-1}$. These peaks are reported to partially arise from citric acid, lactic acid, and acetic acid (Table S5 in the Supplementary Material). The results show a significant decrease in the intensities of these two peaks when *G. vaginalis* is detected both with and without the presence of *L. iners* [Figs. 5(a)–5(b)]. Last, we examined additional biochemical changes when *L. iners* is and is not detected in samples without *G. vaginalis* present ([+*L. iners*, −*G. vaginalis*] versus [*−L. iners*, −*G. vaginalis*]). We found statistical differences among the three peak intensities. First, organic acid content, represented by the intensities at $1610\;{\mathrm{cm}}^{-1}$ and $890\;{\mathrm{cm}}^{-1}$, shows a significant difference when *L. iners* was present and absent when *G. vaginalis* was absent [Figs. 5(a)–6(b)]. Second, the differences in the intensity at $935\;{\mathrm{cm}}^{-1}$ reported to relate to protein, amino acid, and polysaccharide content were investigated (Table S5 in the Supplementary Material). In the absence of *G. vaginalis*, there is a significant increase in this intensity when *L. iners* is not detected in the vaginal fluid samples compared with when it is [Fig. 5(c)].

After establishing that SERS is sensitive to biochemical changes induced by differing bacterial content in vaginal fluid, the use of a portable Raman spectrometer to monitor these biochemical changes was investigated. The SERS spectra of each vaginal fluid solution were collected on a 785 nm Wasatch Photonics portable Raman spectrometer and compared with the spectra collected on the Renishaw Raman microscope. Figure 6 shows the average spectra collected from swab 1 from each participant using both Raman systems. Spectra collected on each system from swab 2 for each participant are shown in Fig. S2 in the Supplementary Material. Small regions of variability are observed for some participants. Similar to before, peak intensity and peak ratio analysis were performed on the spectra collected from the portable system to determine if it can provide adequate spectral information while being more point-of-care friendly.

**Fig. 7:**
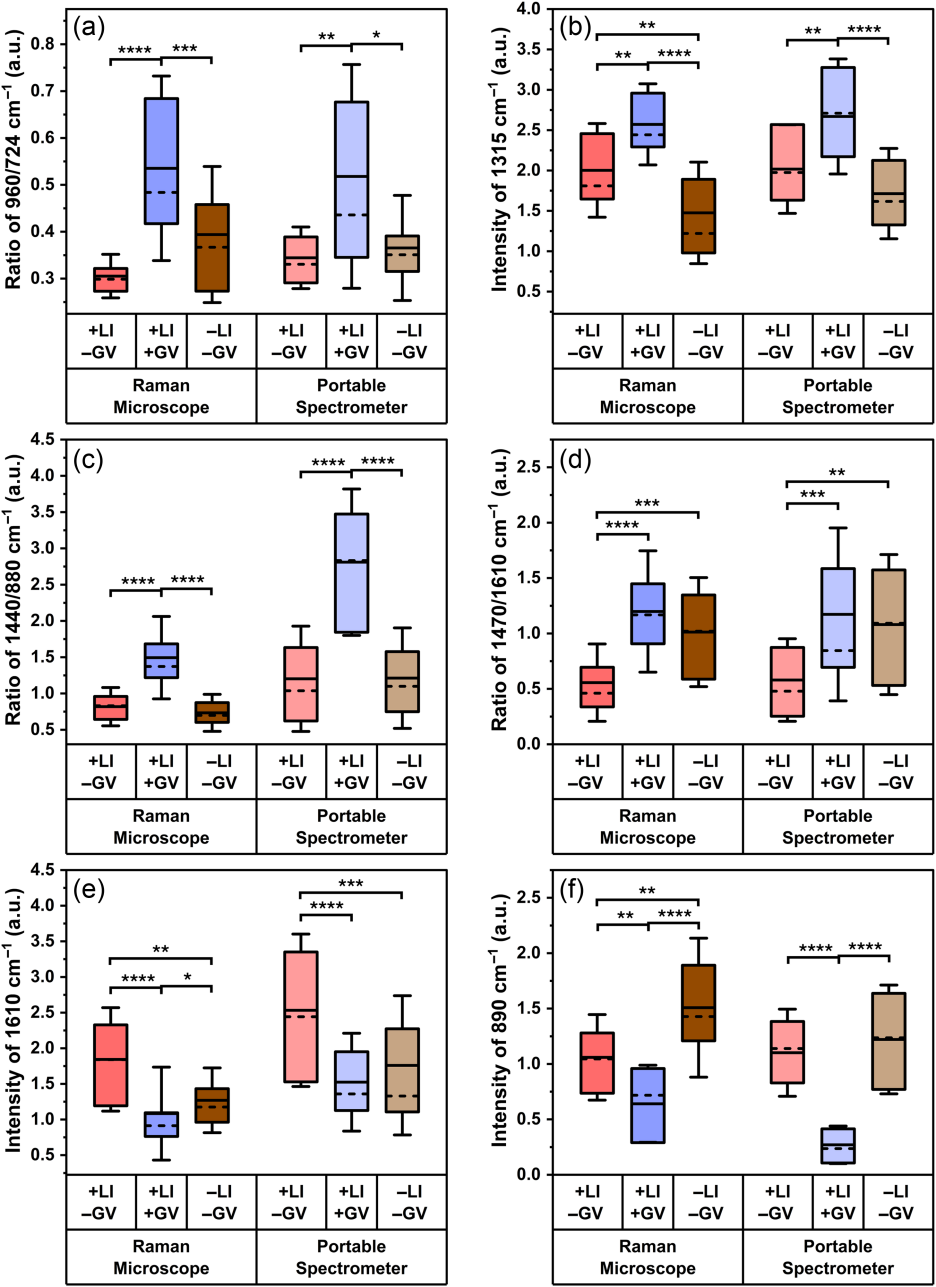
Comparison of peak intensity and peak ratio analysis using the spectra measured on the two Raman spectrometers. (a) Ratio of $960/724\;{\mathrm{cm}}^{-1}$ (protein and lipids/adenine and peptidoglycan); (b) intensity of $1315\;{\mathrm{cm}}^{-1}$ peak (protein, amino acids, and lipids); (c) ratio of $1440/880\;{\mathrm{cm}}^{-1}$ (lipids); (d) ratio of $1470/1610\;{\mathrm{cm}}^{-1}$ (lipids); (e) intensity of $1610\;{\mathrm{cm}}^{-1}$ peak (organic acids); (f) intensity of $890\;{\mathrm{cm}}^{-1}$ peak (organic acids). Box = 25–75 percentile, whiskers = standard deviation, solid line = mean, dashed line = median. +LI/−GV = 14 swabs (n=41 spectra), +LI/+GV = 12 swabs (n=36 spectra), and −LI/−GV = 12 swabs (n=36 spectra). *p<0.05, **p<0.01, ***p<0.001, ****p<0.0001. LI = *L. iners*, GV = *G. vaginalis*.

When examining protein and lipid content in vaginal fluid with differing bacterial content, the peak ratio of $960/724\;{\mathrm{cm}}^{-1}$ and intensity of the $1315\;{\mathrm{cm}}^{-1}$ peak show similar trends when comparing the two systems with equal or minor loss of significance [Figs. 7(a)–7(b)]. For the peak ratio of $960/724\;{\mathrm{cm}}^{-1}$, the [+*L. iners*, +*G. vaginalis*] group measured on the portable system has a decrease in the magnitude of the p-values compared with the Raman microscope but remains significantly higher than the other two groups. The peak intensity at $1315\;{\mathrm{cm}}^{-1}$ has equal levels of significance for the two systems when comparing the [+*L. iners*, +*G. vaginalis*] group to the other two groups, but there is a loss of significance when comparing the [+*L. iners*, −*G. vaginalis*] group with the [−*L. iners*, −*G. vaginalis*] group (microscope: p<0.01, portable spectrometer: p=0.11). Second, when investigating lipid content, the data collected with the portable spectrometer show little-to-no loss in significance as compared with the data collected on the Raman microscope [Figs. 7(c)–7(d)]. Finally, when comparing acid-related peaks from the spectra collected on the two systems, the intensity of $1610\;{\mathrm{cm}}^{-1}$ remains statistically higher for the [+*L. iners*, −*G. vaginalis*] group compared with the other two groups with equal or greater significance. However, there is a loss of significance between the [+*L. iners*, +*G. vaginalis*] and [−*L. iners*, −*G. vaginalis*] group using the portable spectrometer (microscope: p<0.05, portable spectrometer: p>0.05) [Fig. 7(e)]. The second feature chosen to investigate acid-related features was the $890\;{\mathrm{cm}}^{-1}$ peak. This peak intensity shows equal or greater significance when comparing the [+*L. iners*, +*G. vaginalis*] group with the other two groups on the portable spectrometer as compared with the Raman microscope [Fig. 7(f)]. There is a loss of significance when comparing the [+*L. iners*, −*G. vaginalis*] and the [−*L. iners*, −*G. vaginalis*] group on the portable spectrometer (microscope: p<0.01, portable spectrometer: p>0.05).

## 4 Discussion

This work shows that SERS can be utilized to determine the presence of two vaginal microbes, *L. iners* and *G. vaginalis*, in vaginal fluid samples using both a Raman microscope and a portable spectrometer. Overall, we found that the presence of *G. vaginalis* can be characterized by an increase in protein and lipid-related peaks and a decrease in organic acid content. The presence of *L. iners* can be characterized by an increase in protein, amino acid, and polysaccharide-related spectral features. Determining the presence or absence of *L. iners* is clinically important for the administration of appropriate care, as current clinical techniques can often miss *L. iners* in samples*.* In addition, the samples used in this study had *G. vaginalis* concentrations between $10^{2}$ and $10^{4}\;\mathrm{CFU}/\mathrm{mL}$, which is below the concentration typically found during active BV.^24^^,^^50^^,^^51^ Moreover, no participants in this study reported symptoms of BV, and their medical providers did not report findings consistent with a BV diagnosis. Early detection of *G. vaginalis* at sub-infection concentrations could lead to early treatment, thereby mitigating potential negative dysbiosis effects, such as increased risk for acquiring STIs or UTIs.^4^ The detection of these changes before any onset of symptoms reported by the participants shows the promise of SERS in the field for routine vaginal monitoring. Furthermore, previous SERS studies identified spectral changes after infection had been clinically diagnosed; however, this work highlights the potential of SERS for the early detection of *G. vaginalis* prior to clinical diagnosis.

Vaginal fluid is a complex fluid with contributions from numerous biological sources. Vaginal epithelial cells, white blood cells, and microbial cells from the microbiome are components of vaginal fluid. Biochemical constituents include organic acids (lactic, acetic, and citric acid), enzymes, protein and amino acids, lipids (cholesterol and fatty acids), and saccharides (glycogen, glucose, fucose, etc.). Vaginal fluid also contains immunoglobulins and interleukins.^33^^,^^52^^,^^53^ These compounds give rise to the complex SERS spectra of vaginal fluid seen in Fig. 1. All spectra show a strong feature between 723 and $734\;{\mathrm{cm}}^{-1}$, which is commonly reported to be from the bacterial cell wall.^31^^,^^47^^–^^49^ The strength of this peak highlights the high bacterial content found in the collected samples ($10^{7}$–$10^{9}\;\mathrm{CFU}/\mathrm{mL}$).

The low spectral variability observed between the two swabs collected from each participant (Fig. 1) is hypothesized to stem from the sequential collection of the two swabs rather than simultaneous collection. Each swab was collected by a medical provider who was instructed to place the second swab in the same location as the first. There may be natural biochemical heterogeneity within the vaginal environment, which could lead to variability among the sequential swabs. It is known that the microbial content and biochemical composition of vaginal fluid among individuals varies, and this is reflected in the SERS spectra collected here (Fig. 1). These variations in spectral profile may be due to the differing bacteria content in the samples (Table S4 in the Supplementary Material) and multifactorial differences in biochemical composition. The amount of cervical mucus in vaginal fluid has been reported to decrease over the menstrual cycle.^54^ Varying hormone levels, including estrogen, progesterone, luteinizing hormone, and follicle-stimulating hormone, may cause changes to the spectral profile.^27^ Changes in estrogen can alter the abundance of glycogen in vaginal fluid, further varying the spectral profile as estrogen levels change during the menstrual cycle.^55^ This study highlights that SERS is sensitive to these biochemical variations and differences in microbial content.

Spectra collected from each swab were grouped based on the presence and absence of *L. iners* and *G. vaginalis* (Fig. 2). When *L iners* is present without *G. vaginalis*, this group has the lowest coefficient of variation (12.82%). This may indicate that when *L. iners* is present in the vaginal fluid samples, the biochemical composition may be more stable than when it is not present or when present with a pathogen such as *G. vaginalis*. Overall, the PLS results reveal nine spectral regions with a VIP score greater than 1.0 (Fig. 3). Biochemical features associated with these regions are representative of proteins and amino acids, organic acids, polysaccharides, peptidoglycan, and lipids. These molecules are well-known components of vaginal fluid and the bacterial cell wall.^31^^,^^52^

We found that when *G. vaginalis* is present in the vaginal fluid, there is a relative increase in the overall protein and lipid contents (960/724 and $1315\;{\mathrm{cm}}^{-1}$) [Figs. 4(a)–4(b)]. Literature has shown an increase in mucins, large proteins that coat the vaginal epithelium and cervix, and cytokines when *G. vaginalis* is present.^56^^,^^57^ *G. vaginalis* can also produce vaginolysin, a protein-based pore-forming toxin, which may contribute to the increase in protein features.^58^ These two spectral features also relate to lipid content, so to further investigate lipid changes in relation to varying bacterial presence, two additional spectral features were analyzed. The ratio of $1440/880\;{\mathrm{cm}}^{-1}$ shows a significant increase when *G. vaginalis* is present [Fig. 4(c)]. We hypothesize that this increase in lipids could be related to two factors. First, there has been a reported significant increase in short-chain and long-chain fatty acids produced by BV-associated bacteria (i.e., *G. vaginalis*). These fatty acids are theorized to contribute to the development of vaginal dysbiosis.^57^^,^^59^ Second, it is well known that *G. vaginalis* forms biofilms on the vaginal walls, and lipids are a key component of the biofilm matrix (extracellular polymeric substances).^60^^,^^61^ Thus, the increase in lipid content identified in this study could point to *G. vaginalis* beginning to develop biofilms on the vaginal epithelium. We also found that when neither *L. iners* nor *G. vaginalis* are present, there is a significant increase in the lipid-associated ratio of $1470/1610\;{\mathrm{cm}}^{-1}$ as compared with when *L. iners* is present without the co-occurrence of *G. vaginalis* [Fig. 4(d)]. This could stem from the aforementioned increase in fatty acids when dysbiosis-associated bacteria (other than *G. vaginalis*) are present or variations in fatty acid content caused by the differing *Lactobacillus* species, such as *L. gasseri*, dominating the microbiome.^62^

Organic acid content, which is vital to regulating the pH of vaginal fluid, was investigated using the peak intensities at 890 and $1610\;{\mathrm{cm}}^{-1}$.^59^ Both peak intensities are significantly lower when *G. vaginalis* is present [Figs. 5(a)–5(b)]. The significant reduction in these acid-related peaks correlate well with the known increase in vaginal pH that occurs during BV.^59^^,^^63^ Although the *G. vaginalis* concentrations detected in this study were at sub-infection concentrations, the presence of this bacteria could still lead to reductions in organic acids and increases in vaginal pH.

Lastly, we investigated biochemical changes when *L. iners* was and was not present without *G. vaginalis* ([+*L. iners*, −*G. vaginalis*] versus [*−L. iners*, −*G. vaginalis*]). We found significant differences in peak intensities relating to organic acid content. The peak intensity at $1610\;{\mathrm{cm}}^{-1}$ was highest when *L. iners* was present without *G. vaginalis*, whereas the intensity at $890\;{\mathrm{cm}}^{-1}$ was highest when neither *L. iners* nor *G. vaginalis* were present [Figs. 5(a)–5(b)]. Although both of these peaks relate to organic acid content, the $890\;{\mathrm{cm}}^{-1}$ peak is representative of citric acid content, whereas the $1610\;{\mathrm{cm}}^{-1}$ peak relates to lactic and acetic acid (Table S5 in the Supplementary Material). These differences in biochemical peak identifications may be why differing patterns are seen when examining these features. *L. iners* is not known to produce bacteriocins, an antimicrobial peptide, yet the other common vaginal *Lactobacillus* can produce these molecules.^64^ When *L. iners* was not detected in the vaginal fluid, there is a trend of increasing intensity of the $935\;{\mathrm{cm}}^{-1}$ peak [Fig. 5(c)]. This peak has been reported to arise from protein, amino acids, and polysaccharides (Table S5 in the Supplementary Material). The production of bacteriocins by the other *Lactobacillus* species could increase the relative amino acid-associated SERS peaks. In addition, *L. iners* is unable to produce cysteine and must acquire it from its environment.^65^ The removal of cysteine from the environment could also result in alterations to the amino acid content in the vaginal fluid, which could cause the spectral variation seen. Finally, *L. iners* is correlated with increased fucosylation, a type of glycosylation in which fucose sugars are added to molecules.^14^^,^^66^ This increase in fucosylation could alter the amount of saccharides in the vaginal fluid, increasing the signals from mono- and polysaccharides. Moreover, these results demonstrate that SERS can be used not only to detect pathogenic species such as *G. vaginalis* but also to detect differences relating to the presence of *Lactobacillus* species.

A portable Raman spectrometer enhances the potential for clinical translation of this detection technique as this system is more point-of-care friendly. To determine if the biochemical differences measured using a benchtop microscope were feasible to acquire on a portable Raman spectrometer, the spectra of each sample collected with the two systems were compared. Small regions of spectral variability are seen between the two systems, as shown in Fig. 6. These regions of variability may be due to differences in laser spot sizes between the two systems. The laser spot under the 20× objective on the Raman microscope is a rectangular beam with an approximate length of 56  μm and a width of 9  μm. The Wasatch portable spectrometer has a circular beam profile with a diameter of 168  μm. As the excited area is ∼44 times larger for the portable spectrometer, this could result in different molecules being represented in the spectra compared with those on the microscope system. In addition, the two systems have different spectral resolutions: $\text{1 }{\mathrm{cm}}^{-1}$ for the Raman microscope and $7\;{\mathrm{cm}}^{-1}$ for the portable spectrometer.^67^^,^^68^ The difference in spectral resolution could have affected the shape of certain peaks, resulting in the spectral variation observed in Fig. 6. Regardless of these differences, the portable spectrometer can identify biochemical changes in vaginal fluid based on bacterial presence, similar to the benchtop microscope (Fig. 7). Similar levels of significance were found when comparing peak intensities and ratios calculated from the spectra collected on each Raman system. These results show that SERS detection of vaginal microbiota can be performed on a lower cost portable system negating the need for a bulky Raman microscope.

Limited studies have been conducted using SERS to study the biochemical profile of vaginal fluid.^27^^,^^33^^–^^35^ These previous studies utilized flawed clinical standards, such as the Nugent method, to determine the bacteria present in collected samples. This study benefits from the use of qPCR for the accurate determination of bacterial presence in our samples. This is also the first study to show the spectral profile of vaginal fluid with a gold plasmonic substrate, as all previous studies utilize silver. Finally, this study benefits from a comparative analysis of spectra collected using a benchtop microscope and portable Raman spectrometer, thereby highlighting the clinical translatability of this technique. This pilot study aids in expanding the breadth of research into the use of SERS for detecting biochemical differences and microbial content in vaginal fluid. As this work is a pilot study, it is limited by a modest sample size, and future work will focus on expanding the sample size and demographics. In addition, only four bacterial species were measured via qPCR in this study, limiting the full characterization of the vaginal microbiome. Future work will incorporate 16S rRNA gene sequencing for full microbiome analysis and comparison with the SERS spectra. Furthermore, although *L. crispatus* was detected in some of the samples utilized here, there was not a sufficient number of *L. crispatus* samples to allow for the stratification of the data to fully investigate the impact of this species on the spectral profile. As additional samples are collected for continuing analyses, the impact of *L. crispatus* will be further investigated. Overall, this study highlights the strength of SERS for clinical monitoring of bacterial presence in vaginal fluid.

## 5 Conclusion

In this pilot study, the SERS spectra of vaginal fluid samples were characterized and used to determine biochemical changes in relation to bacterial presence. Significant changes were found when *L. iners* was present with and without *G. vaginalis*, and when *L. iners* was not detected in the vaginal fluid samples. By determining the presence of specific bacterial species, SERS can help overcome the limitations of current clinical diagnostic techniques, such as the Nugent method, which may fail to detect *L. iners*. Furthermore, the use of a portable Raman spectrometer for vaginal fluid analysis showed results comparable to a conventional Raman microscope, highlighting the point-of-care potential of this technique. Overall, this study shows the potential of SERS to detect bacterial species in the vaginal microbiome toward increased monitoring of vaginal health.

## Supplementary Material

10.1117/1.BIOS.2.4.042102.s01

## Data Availability

Data, code, figures, and materials will be made available upon reasonable request.
