# Supplementary material for: Investigating microbiota and biochemical changes in vaginal fluid toward point-of-care microbial monitoring using surface-enhanced Raman spectroscopy
Source: Biophotonics Discov. 2025 Jul 25;2(4):042102. doi: 10.1117/1.BIOS.2.4.042102 (PMC13101066; doi:10.1117/1.BIOS.2.4.042102)
Supplement: Supplementary file 1 [file BIOS_002_042102_SD001.pdf]

Supplementary Materials

Bacteria culturing

L. iners was cultured from frozen stock on NYC III agar plates at 37°C under 5% CO_2 for 24 hours. Following the colony growth, a liquid culture was grown by transferring a single colony to 5 mL of NYC III broth and was allowed to incubate for 24 hours (37 °C and 5% CO_2). *G. vaginalis* was cultured using NYC III media and incubated for 48 hours at 37 °C and 5% CO_2. *L. crispatus* was cultured using De Man–Rogosa–Sharpe (MRS) media for 24 hours at 37 °C and 5% CO_2. *S. agalactiae* was cultured on brain heart infusion (BHI) agar for 24 hours at 37°C under 5% CO_2. All cultures were then washed with sterile deionized (DI) water twice by centrifugation at 3300 relative centrifugal force (RCF) for 8 min, and 1 mL of washed culture was collected for optical density (OD) readings at 600 nm to quantify the bacterial concentration using UV-vis-NIR spectrophotometry.

Variable	n or mean (range)		p-value
Age (yr)	40.0 (26-60)		0.46
Race/Ethnicity	Caucasian	15	0.26
	African American	4	
Sexual intercourse with in 2 weeks	Yes	8	0.27
	No	11	
Contraceptive use	Yes	9	0.12
	No	9	
	Unknown	1	

Table S1. Participant demographics and p-value from Kruskal-Wallis test showing no significant differences in participant demographics between the three assigned groups.

	Raman Microscope	Portable Spectrometer
**System Name**	Renishaw inVia Qontor Raman Microscope	Wasatch WP-785X-F18-R-ILC portable Raman spectrometer
**Excitation Wavelength**	785 nm	785 nm
**Beam Profile**	Rectangular<br>56 µm x 9 µm	Circular<br>168 µm diameter
**Spectral Resolution**	1 cm^{-1}	7 cm^{-1}
**Measurement Configuration**	20X microscope objective<br>NA = 0.4<br>working distance=1.15 mm<br>1200 l/mm grating	Fiber optic probe<br>105 µm core diameter<br>NA = 0.22<br>working distance = 22 mm<br>f/1.8
**Detector Specifications**	Deep-depleted CCD at -70°C<br>1024 x 256 pixels<br>26 x 26 µm pixel size	Hamamatsu back-thinned CCD at 10°C<br>2048 x 64 pixels<br>14 x 14 µm pixel size

Table S2. Specifications of each Raman system utilized.

Bacteria	Primer Name	Sequence	Working Con.	Cycling Conditions	Ref.
L. iners	InersFw	5′-GTCTGCCTTGAAGATCGG-3′	200 nM	10' 95°C, (1' 95°C, 1' 55°C, 1' 65°C) × 45	21
	InersRev	5′-ACAGTTGATAGGCATCATC-3′	200 nM		
G. vaginalis	F-GV1	5′-TTACTGGTGTATCACTGTAAGG-3′	200 nM	10' 95°C, (45" 94°C, 45" 55°C, 45" 72°C) × 45	
	R-GV3	5′-CCGTCACAGGCTGAACAGT-3′	200 nM		
L. crispatus	LcrisF	5’-AGCGAGCGGAACTAACAGATTTAC-3’	100 nM	10' 95°C, (15" 95°C, 1’ 60°C) × 45	
	LcrisR	5’-AGCTGATCATGCGATCTGCTT-3’	100 nM		
S. agalactiae	Sag59	5′-TTTCACCAGCTGTATTAGAAGTA-3′	200 nM	10' 95°C, (10" 95°C, 14" 60°C, 20" 72°C) × 45	39
	Sag190	5′-GTTCCCTGAACATTATCTTTGAT-3′	200 nM		

Table S3. Primer name, sequence, working concentration, and cycling conditions for qPCR assays.

Participant Number	Swab Number	*L. iners* Concentration	*G. vaginalis* Concentration	*L. crispatus* Concentration	Assigned Group
1	1	4.01×10^7	*	*	1) +*L. iners*, -*G. vaginalis*
	2	4.89×10^7	*	*	1) +*L. iners*, -*G. vaginalis*
2	1	*	*	5.44×10^6	3) -*L. iners*, -*G. vaginalis*
	2	*	*	1.97×10^6	3) -*L. iners*, -*G. vaginalis*
3	1	*	*	*	3) -*L. iners*, -*G. vaginalis*
	2	*	*	*	3) -*L. iners*, -*G. vaginalis*
4	1	2.37×10^8	*	*	1) +*L. iners*, -*G. vaginalis*
	2	7.43×10^7	*	*	1) +*L. iners*, -*G. vaginalis*
5	1	1.14×10^8	9.05×10^4	*	2) +*L. iners*, +*G. vaginalis*
	2	1.79×10^8	1.08×10^5	*	2) +*L. iners*, +*G. vaginalis*
6	1	1.30×10^8	*	*	1) +*L. iners*, -*G. vaginalis*
	2	3.08×10^8	*	*	1) +*L. iners*, -*G. vaginalis*
7	1	*	*	*	3) -*L. iners*, -*G. vaginalis*
	2	*	*	*	3) -*L. iners*, -*G. vaginalis*
8	1	*	*	4.64×10^7	3) -*L. iners*, -*G. vaginalis*
	2	*	*	7.85×10^7	3) -*L. iners*, -*G. vaginalis*
9	1	5.03×10^6	3.15×10^4	*	2) +*L. iners*, +*G. vaginalis*
	2	1.02×10^6	7.86×10^3	*	2) +*L. iners*, +*G. vaginalis*
10	1	1.76×10^7	9.55×10^3	*	2) +*L. iners*, +*G. vaginalis*
	2	5.29×10^7	1.05×10^4	*	2) +*L. iners*, +*G. vaginalis*
11	1	8.31×10^7	*	*	1) +*L. iners*, -*G. vaginalis*
	2	7.69×10^7	*	*	1) +*L. iners*, -*G. vaginalis*
12	1	1.09×10^4	*	7.44×10^7	1) +*L. iners*, -*G. vaginalis*
	2	1.13×10^4	*	1.48×10^8	1) +*L. iners*, -*G. vaginalis*
13	1	3.87×10^6	*	*	1) +*L. iners*, -*G. vaginalis*
	2	1.39×10^6	*	*	1) +*L. iners*, -*G. vaginalis*
14	1	2.03×10^7	5.16×10^4	*	2) +*L. iners*, +*G. vaginalis*
	2	5.49×10^7	9.81×10^4	*	2) +*L. iners*, +*G. vaginalis*
15	1	1.81×10^8	8.11×10^2	*	2) +*L. iners*, +*G. vaginalis*
	2	1.71×10^8	9.65×10^2	*	2) +*L. iners*, +*G. vaginalis*
16	1	1.13×10^7	*	1.40×10^6	1) +*L. iners*, -*G. vaginalis*
	2	2.87×10^7	*	1.67×10^7	1) +*L. iners*, -*G. vaginalis*
17	1	*	*	2.10×10^7	3) -*L. iners*, -*G. vaginalis*
	2	*	*	1.24×10^7	3) -*L. iners*, -*G. vaginalis*
18	1	*	*	*	3) -*L. iners*, -*G. vaginalis*
	2	*	*	*	3) -*L. iners*, -*G. vaginalis*
19	1	1.07×10^8	1.25×10^2	*	2) +*L. iners*, +*G. vaginalis*
	2	7.19×10^7	6.61×10^2	*	2) +*L. iners*, +*G. vaginalis*

Table S4. Bacterial concentrations (CFU/mL) for each swab as determined by qPCR and assigned group (right-most column). *indicates this bacteria was not detected in the sample.

Raman Peak (cm^{-1})	Vibrational mode	Tentative biochemical assignment	Refs.
622	COO^- wagging, C-C twist	Amino acids	69,72
650	ν(C-S), δ(COO^-), C-C twist	Amino acids	27, 69, 71
685	δ(-C-H)	Protein	71
723-734	Ring breathing mode of adenine, C-C twist, ν(C-S)	Adenine, peptidoglycan	27
850	Fermi resonance between ring breath, out-of-plane ring bend overtone, ν(C-C), benzene ring breathing, ν(C-O-C)	Amino acids, acetic acid, lactic acid	27, 71, 72
880-890	ν(C-C)	Protein, glucose, citric acid	27, 33, 69, 71
920	ν(C-C), CH_3 vibrations	Lactic acid, amino acids	70, 72
935	ν(C–COO^-), δ_{sym}(-C-C-N), ν(C-C)	Protein, amino acids, polysaccharides	27, 71, 72
950-960	$\nu_{skeletal}$(N-C-C)	Protein, amino acids, lipids polysaccharides	27, 33, 70, 71
1000	Benzene ring breathing, ν(C-N)	Amino acids, urea	27, 69, 71, 72
1028	ν(C-N), ν(C-C), C-H in-plane bending	Phospholipids, carbohydrates, amino acids	69, 72
1085	CO vibrations	Lactic acid	70
1175	δ(NH_2^+), ν(-C-N), C-H bending	Amino acids, protein, nucleic acids	27, 71, 72
1240	Amide III	Protein	27, 69
1290	CH_2 wagging, Amide III, δ_{bend}(N-H), CH_2 twist	Protein, amino acids, lipids, fatty acids	27, 33, 71, 72
1305-1315	CH_2 wagging	Protein, amino acids, lipids	34, 49, 72
1360	CH_3 symmetric bend	Amino acids	72
1440-1450	CH_2 sciss, δ(CH_2), δ(CH_3), COH bending, ν(C-H)	Lipids, protein, amino acids, glucose	33, 49, 69, 71, 72
1470	δ(CH_2), CH_2 bending, CH_2 wagging, C-H vibrations	Protein, lipids, phospholipids	49, 69
1575	NH_2 sciss, ν(benzene ring), ν(pyrrole ring), δ(C=C), ν(C=O)	Protein, amino acids, nucleic acids	49, 71
1595-1640	CH_3, C=O, ν(benzene ring), ν_{asym}(COO^-), ν(C=C)	Protein, amino acids, lactic acid, acetic acid	27, 33, 35, 70, 71

Table S5. Vibrational modes and tentative biochemical assignments for peaks identified in SERS spectra of vaginal fluid. (ν= stretching, δ=deformation, twist = twisting, sciss=scissoring, sym= symmetric, asym= asymmetric)

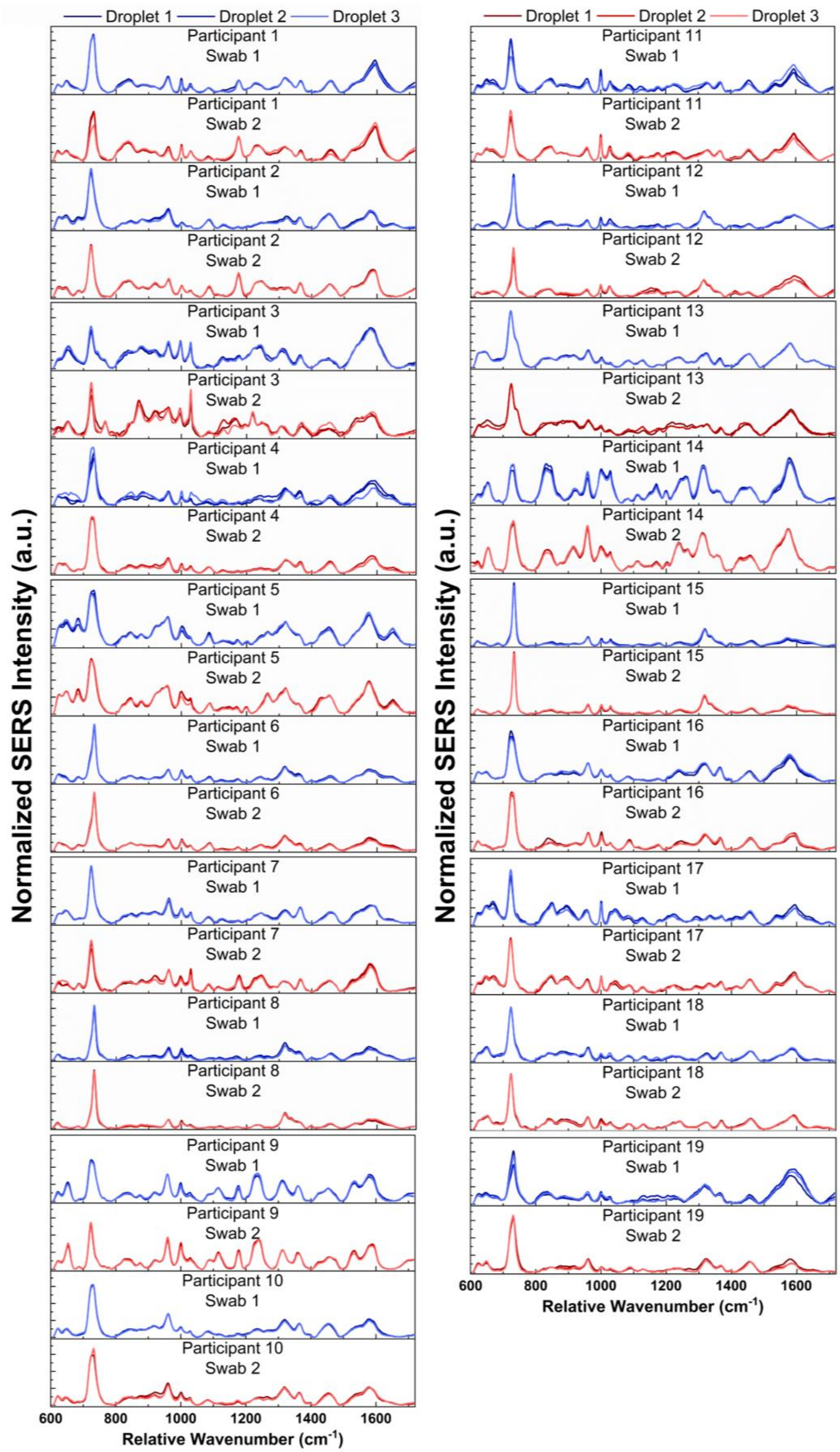

Figure S1. Average spectra (of 5 measurements) collected from each droplet from each vaginal fluid sample collected.

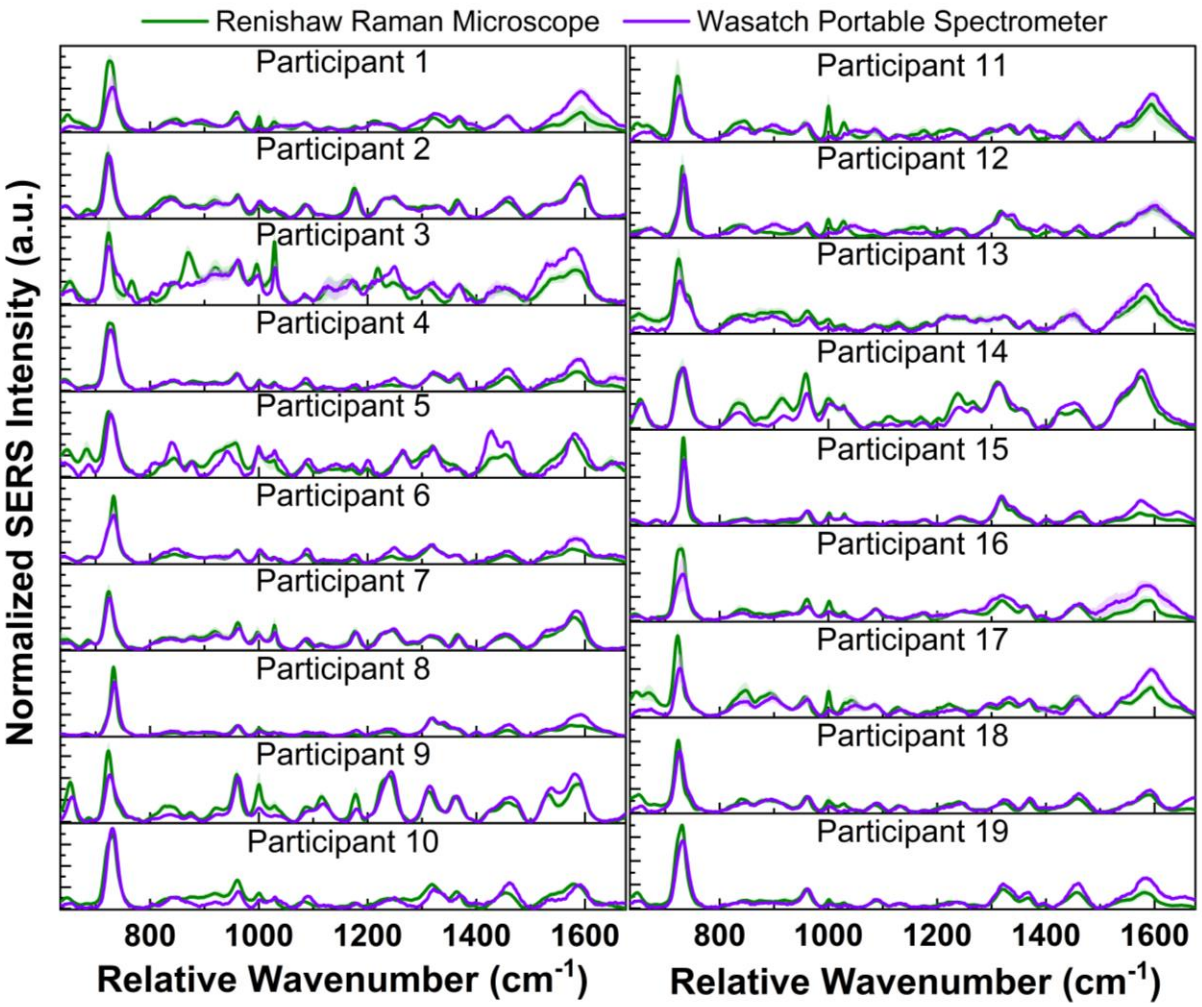

Figure S2. Comparison of vaginal fluid spectra collected from each participant using the Renishaw Raman microscope (green line) and Wasatch portable spectrometer (purple line). Each spectrum is the mean and standard deviation (represented as shaded error bars) for the 3 droplets measured from Swab 2 from each participant (n=15 spectra/swab).
